# Equity of access in rural and metropolitan dementia diagnosis, management, and care experiences: an exploratory qualitative study

**DOI:** 10.1186/s12939-025-02434-1

**Published:** 2025-03-17

**Authors:** Hannah Gulline, Sarah Carmody, Mark Yates, Amelia Bevins, Amy Brodtmann, Samantha M. Loi, Yen Ying Lim, Heather Macklin, Karen Glennen, Michael Woodward, Scott Ayton, Darshini Ayton

**Affiliations:** 1 Health and Social Care Unit, School of Public Health and Preventive Medicine, Faculty of Medicine, Nursing, and Health Sciences, Monash University, Victoria, Australia; 2 Ballarat Clinical School, School of Medicine, Faculty of Health, Deakin University, Victoria, Australia; 3 Grampians Health, Ballarat, VIC Australia; 4 Department of Neuroscience, School of Translational Medicine, Monash University, Victoria, Australia; 5 Eastern Cognitive Disorders Clinic, Eastern Health Clinical School, Monash University, Victoria, Australia; 6 Department of Neurology, Royal Melbourne Hospital, Melbourne, VIC Australia; 7 Florey Institute of Neuroscience and Mental Health, Melbourne, VIC Australia; 8 Neuropsychiatry Centre, Royal Melbourne Hospital, Melbourne, VIC Australia; 9 Department of Psychiatry, University of Melbourne, Victoria, Australia; 10 School of Psychological Sciences, Turner Institute for Brain and Mental Health, Monash University, Victoria, Australia; 11 Lived Experience Expert, North-West Victoria, Australia; 12 Lived Experience Expert, South-West Victoria, Australia; 13 Aged and Continuing Care Services, Austin Health, Victoria, Australia; 14 University of Melbourne, Victoria, Australia; 15 Florey Department of Neuroscience and Mental Health, University of Melbourne, Victoria, Australia

**Keywords:** Health equity, Dementia, Rurality, Access

## Abstract

**Background:**

The limited allocation of resources to rural and regional communities is a major contributor to healthcare inequities in Australia. Distribution of health service resources between metropolitan and rural communities commonly sees highly populated areas prioritised over more sparsely populated and geographically vast areas. As such, challenges impacting dementia diagnosis, management, and care in metropolitan areas are experienced more acutely in rural areas. This study aimed to examine equity of access to dementia diagnosis, management, and care services amongst people who experienced the process of dementia diagnosis as a patient or significant other (partner/spouse, adult children, siblings, and friends) throughout rural and metropolitan Australia.

**Methods:**

This exploratory qualitative study consisted of thirty-three online semi-structured interviews with thirty-seven people with experience of the dementia diagnosis process as a patient and/or significant other. Interviews explored symptoms of dementia, health professionals consulted, tests conducted, and challenges faced throughout the diagnosis and post-diagnosis process. Rurality was defined by the Australian Statistical Geography Standard Remoteness Areas (ASGS-RA) and the Modified Monash Model (MMM). Thematic analysis was conducted, with Russell’s (2013) Dimensions of Access framework (geography, affordability, availability, acceptability, accommodation, awareness, and timeliness) guiding data analysis.

**Results:**

Participants were distributed across various regions of Australia: seven interviews from inner regional Australia, five interviews from outer regional Australia, and twenty-one interviews from metropolitan areas. Disparities in access between metropolitan and rural areas emerged in five key dimensions: 1) geography impeding ability to access services; 2) affordability of travel expenses; 3) availability of healthcare and support services; 4) acceptability of available health professionals and services; and 5) awareness of local services and resources. The dimensions of accommodation and timeliness of care were experienced as challenges irrespective of location, with lengthy appointment wait times and difficulty navigating complex systems. However, rurality often compounded the challenges in dementia diagnosis, management, and care.

**Conclusions:**

Significant health inequities persist between rural and metropolitan communities that must be prioritised in endeavours to promote equitable dementia diagnosis, management, and care. Targeted action to address disparities is vital to mitigate the impact of rurality, particularly as clinical practice evolves with research advancements.

**Supplementary Information:**

The online version contains supplementary material available at 10.1186/s12939-025-02434-1.

## Background

The availability and distribution of health professionals and services amongst geographically diverse communities is one of the major causes of health inequity [1]. Approximately half of the world’s population reside in rural areas, yet there remains uneven distribution of health service resources between metropolitan and rural communities [2]. Fewer than a quarter of the health physician workforce practice in rural areas globally [3]. In Australia, this rural-metropolitan healthcare dilemma is further magnified due to the sparsely populated and vast geographical expanse of the country [4, 5]. The United States in comparison, is also geographically vast and is around 1.28 times larger than the size of Australia, yet it has approximately 12.60 times the population [6].

The scarcity and spread of health resources in rural communities are substantial challenges to achieving equitable dementia diagnosis, management, and care, with health service workforce predicaments amplified in rural regions [7]. Rural communities globally report having fewer primary care physicians, specialists, and support services when compared to their metropolitan counterparts, which greatly limits their access to essential health services and support [1, 7]. For many rural residents, the limited medical infrastructure and dementia workforce necessitates considerable travel distances to access the required care. These long travel distances to access care in larger cities is further compounded by limited affordable and convenient transportation alternatives [8, 9]. The broad, generalist roles of many rural health professionals often mean they lack comprehensive training in dementia. Studies have shown that many rural community-based health professionals report needing additional dementia-specific education to enhance their knowledge and skills in diagnosis, management, and care [10]. Additionally, geographically detached health infrastructure, service disintegration due to remoteness, and workforce shortages present significant barriers for dementia health and social care [11, 12]. Conversely, rural communities can foster supportive, long-term relationships that share caregiving responsibilities and provide practical and emotional support, enhancing the sense of security for people with dementia and their families [13].

The intersectionality of inequity in rural communities underscores the complex interplay of various social determinants that collectively influence health outcomes [14, 15]. In rural communities, factors such as socio-economic status, education, ethnicity, and gender intersect to shape health disparities [14, 15]. These intersecting inequalities are often intensified by the distinct challenges of rural life, including geographical isolation, limited healthcare access, and economic deprivation [15]. It is essential to consider geographical inequalities beyond area-level deprivation, incorporating both contextual and compositional factors to fully grasp the multifaceted nature of health inequities in rural communities [15].

Dementia care in Australia, delivered through a mix of public and private funding, is marked by significant variability in diagnostic approaches, assessment procedures, clinical care quality, and service accessibility [16–18]. Diagnosis, management, and care are administered across a spectrum of settings, including primary care practices, community health services, hospitals, specialized multidisciplinary memory clinics, private specialist practices (such as those of neurologists and geriatricians), and residential aged care facilities [18]. A priority of the National Dementia Action Plan is to promote equity and human rights, ensuring everyone, including those in rural areas, has equitable access to quality healthcare and supports [19].

Dementia diagnosis, management, and care is on the verge of a new epoch, with advances in diagnostic testing, symptom management medications, and, potentially, disease-modifying therapies [20, 21]. These advancements provide an opportunity to shift the dementia care paradigm and rethink current practices that pose significant challenges for rural and remote residents. Prioritising the influence of rurality on dementia care pathways is vital to prevent these innovations from contributing to dementia inequity and broadening the gap between rural and metropolitan communities. The current dementia care paradigm must be understood in order to create the new paradigm. The aim of this paper is to examine equity of access to dementia diagnosis, management, and care for people who experienced the dementia diagnosis process as a patient or significant other (partner/spouse, adult children, siblings, and friends) in the context of rural and metropolitan locations in Australia.

## Methods

### Design

An exploratory qualitative study [22] was conducted to gain insight into the experiences of dementia diagnosis, management, and care of people with dementia and significant others living in metropolitan and rural Australia. A Consumer Advisory Committee was established as part of broader research into dementia diagnosis in Australia to leverage the experiences and expertise of people living with dementia and significant others. While research experience was not a prerequisite to participant within this committee, all members of this group have shared their lived experience with other research studies. This Consumer Advisory Committee, consisting of five people living with dementia and four significant others (three adult children and one partner/spouse), advised the development of the qualitative study. Five of these nine consumers live in rural Australia and hence have lived experience in rural dementia diagnosis, management, and care. This paper compares findings from participants living in regional and rural Australia with those living in metropolitan areas. This paper is reported in compliance with the Consolidated Criteria for Reporting Qualitative Studies (COREQ) 32-item checklist [23].

### Recruitment and Sampling

Participants were recruited through social media advertisements and purposive sampling [24] was employed through consumer organisations such as Dementia Australia and research studies known to the authors including the Healthy Brain Project [25] and the BetterBrains Randomised Controlled Trial [26]. Consumer Advisory Committee members were also invited to participate and snowball sampling [24] was employed, with some members sharing the invitation with other individuals who met the eligibility criteria. People who had experienced the dementia diagnosis process in Australia were eligible to participate in the study if they were over 18 years of age and were able to provide informed consent or assent prior to participation.

Each participant’s rurality was assessed using the Australian Statistical Geography Standard Remoteness Areas (ASGS-RA) and the Modified Monash Model (MMM). The ASGS-RA categorises regions based on geographic access to services, using the Accessibility/Remoteness Index of Australia Plus (ARIA +) [27, 28]. This system divides Australia into five remoteness classes: Major Cities, Inner Regional, Outer Regional, Remote, and Very Remote. Meanwhile, the MMM classifies locations into seven classes, from metropolitan (MM1) to very remote (MM7), based on remoteness and population size, influencing the distribution of health workforce and resources [29, 30].

### Data collection

Semi-structured interviews were conducted online using Zoom (Zoom Video Communications, Inc), with the option of phone calls to support participation if necessary. The Consumer Advisory Committee advised on the interview guide. Interview questions explored the symptoms noticed, health professionals consulted, diagnostic tests performed, and communication between health professionals and patients/significant others. Each participant discussed beneficial elements, areas requiring improvement, and recommendations for change to the pre-diagnosis, diagnosis and post-diagnosis experience. The interview guide was tailored according to whether the interview was with a person who experienced the diagnosis process as a patient and/or a significant other, with both versions available in Additional files 1 and 2. Each participant was provided with the interview guide prior to the interview. Data collection was conducted from July 2022 to June 2023. This study, conducted to inform broader research into dementia diagnosis, concluded recruitment after collecting sufficient data from diverse participant experiences to enable the progression to the next research phase [31].

The interview guide was pilot tested with members of the Consumer Advisory Committee and adjustments were made to enhance exploration of diagnosis experiences. Interviews were conducted by SC, HG, and/or DA who are all trained in public health and qualitative research methods. None of the interviewers were clinically trained or were involved in the clinical care of the participants.

### Data analysis

Interviews were audio-recorded and transcribed, with a qualitative data analysis program (QSR-NVivo) used to manage transcriptions for analysis. A coding guide was developed from the interview guide by SC and inductive coding was used as new themes were identified by SC, HG, and DA [32]. Equity of access was identified as a central theme, in particular, differentiating experiences between rural and metropolitan diagnosis journeys.

To further explore equity of access, the Dimensions of Access framework by Russell et al. [33] was applied to the data. Russell et al. [33] disaggregated the concept of access into seven dimensions to facilitate policy-makers to develop and evaluate primary health care policies to bridge the gap between metropolitan cities and rural areas. This framework dissects the complex concept of access into distinct dimensions, facilitating the examination of contextual and compositional factors of rurality such as resources and services, infrastructure, socio-economic status, health literacy, and socio-cultural needs. The framework dimensions of geography, affordability, availability, acceptability, accommodation, awareness, and timeliness guided analysis of accessibility in rural dementia diagnosis journeys. Interviews were initially coded inductively to identify key experiences of diagnosis, management, and care [32]. A second round of deductive coding was employed to map data to the seven dimensions of access by HG, with DA and SC consulted on each code [32]. Findings were presented back to the Consumer Advisory Committee. Two members of the Consumer Advisory Committee who have experienced dementia diagnosis, management and care in rural communities reviewed the paper and provided feedback (HM and KG).

### Ethical considerations

Ethics approval for this study was received from the Monash University Human Research Ethics Committee (Project ID 34523). Each participant provided verbal or written informed consent before participating in an interview. Study participation was voluntary, with each participant informed of their right to withdraw consent at any point or refuse to answer a question. Each participant was offered a $50 voucher for their participation in the study. Pseudonyms were assigned to ensure each participant’s anonymity and all data was de-identified.

## Results

Thirty-three interviews (37 participants – 4 were dyad interviews) were conducted with participants from different regions of Australia: seven interviews from inner regional Australia (9 participants), five interviews from outer regional Australia (5 participants), and twenty-one interviews from metropolitan areas (23 participants). Participants were people who experienced dementia diagnosis as a patient (rural *n* = 6, metropolitan *n* = 5) and significant other (rural *n* = 8, metropolitan *n* = 18; see Table 1). Interviews ranged in length from 40–75 min.

**Table 1:** Participant demographics

**Demographic Characteristics**	**Rural (*n*** **= 14) n (%)**	**Metropolitan (*n*** **= 23) n (%)**
Participant Type		
Person undergone diagnosis as patient	6 (42.9)	5 (21.7)
Adult Child	6 (42.9)	11 (47.8)
Partner/Spouse	1 (7.1)	6 (26.1)
Other – Sibling, Friend	1 (7.1)	1 (4.3)
Sex		
Male	2 (14.3)	7 (30.4)
Female	12 (85.7)	16 (69.6)
**Diagnosis Characteristics**	**Rural (*n* = 12)**	**Metropolitan (*n* = 21)**
Diagnosis		
Alzheimer’s	4 (33.3)	9 (42.8)
Frontotemporal Dementia	2 (16.7)	4 (19.0)
Lewy Body Dementia	0 (0.0)	1 (4.8)
Vascular Dementia	2 (16.7)	1 (4.8)
Mixed Dementia (Alzheimer’s and Vascular)	1 (8.3)	2 (9.5)
Dementia (subtype not reported)	1 (8.3)	2 (9.5)
Mild Cognitive Impairment	1 (8.3)	1 (4.8)
No Dementia/Mild Cognitive Dementia Diagnosis	1 (8.3)	1 (4.8)
Age at Diagnosis		
≤ 65 years	5 (41.7)	4 (19.0)
66 – 75 years	3 (25.0)	4 (19.0)
76 – 85 years	1 (8.3)	3 (14.3)
> 85 years	0 (0.0)	3 (14.3)
Not reported	2 (16.7)	6 (28.6)
No Dementia/Mild Cognitive Dementia Diagnosis	1 (8.3)	1 (4.8)
Years Since Diagnosis		
Diagnosis 0–5 years	6 (50.0)	8 (38.1)
Diagnosis 6–10 years	2 (16.7)	7 (33.3)
Diagnosis 10 + years	3 (25.0)	3 (14.3)
No diagnosis	1 (8.3)	1 (4.8)
Not reported	0 (0.0)	2 (9.5)
State/Territory of Diagnosis		
Victoria	6 (50.0)	8 (38.1)
Queensland	2 (16.7)	6 (28.6)
Western Australia	1 (8.3)	2 (9.5)
New South Wales	1 (8.3)	2 (9.5)
South Australia	1 (8.3)	2 (9.5)
Tasmania	1 (8.3)	0 (0.0)
Australian Capital Territory	0 (0.0)	1 (4.8)
Modified Monash Model^a^		
MM1	-	21 (100.0)
MM2	5 (41.7)	-
MM3	3 (25.0)	-
MM4	3 (25.0)	-
MM5	1 (8.3)	-
Australian Statistical Geography Standard Remoteness Areas^b^		
Major Cities of Australia	-	21 (100.0)
Inner Regional Australia	7 (58.3)	-
Outer Regional Australia	5 (41.7)	-

^a^Modified Monash Model: MM1—Metropolitan; MM2—Regional centres; MM3—Large rural towns; MM4—Medium Rural Towns; MM5—Small Rural Towns; MM6—Remote communities; MM7—Very remote communities [29]

^b^ Australian Statistical Geography Standard Remoteness Areas: Major Cities of Australia; Inner Regional Australia; Outer Regional Australia; Remote Australia; Very Remote Australia [28]

Equity in access was the foremost difference in the experiences of rural and metropolitan Australians navigating dementia diagnosis, management, and care. Applying the Dimensions of Access framework [33] to the data further explored the different aspects of access experienced throughout the rural and metropolitan journeys. The themes mapped to the seven dimensions are outlined in Table 2.

**Table 2:** Dimensions of Access [33] definitions and themes

Dimension	Themes
***Geography*** *Ease and ability by which a person can transcend the distance to a healthcare service*	The added challenge of residing far from health and dementia specialist services Visiting specialists to rural communities significantly reduced travel burden Accessing healthcare and dementia specialist services virtually reduced geographical burden Limited viable transport alternatives in rural communities
***Affordability*** *Ease by which a person can settle the total costs for needed health care*	Greater perceived costs associated with travel to various appointments and tests when compared to metropolitan areas
***Availability*** *Types and volumes of healthcare services and facilities in regard to the population’s healthcare needs*	Limited access and availability to healthcare and support services in rural communities Disparity in access and choice to healthcare services and support in rural communities
***Acceptability*** *Consumer attitudes and beliefs about their health to the personal and practice characteristics of providers*	Lower expectations of healthcare and services due to limited local options in rural communities
***Accommodation*** *Organisation of healthcare services in regards to a person’s ability to contact, enter and navigate the system to meet healthcare needs*	Rural location often compounded the complexity to navigating health and social care systems
***Awareness*** *The communication of health and health system information between health services and consumers*	Insufficient knowledge of accessible services in rural communities led to limited post-diagnostic support
***Timeliness*** *Extent to which individuals seek and receive healthcare within a time frame they consider optimal for achieving the greatest health outcomes*	Relatively long waiting times for appointments with specialists and tests for rural and metropolitan areas

### Geography

Travelling long distances was a major challenge to accessing healthcare and dementia specialist services. Participants classified as living in outer regional Australia most commonly described the added challenge of residing far from health and dementia specialist services, requiring lengthy travel to larger towns and cities. Several participants in outer regional Australia described travelling 200–550 kms to access dementia diagnosis services, although two lived in regional centres with local services available. One daughter noted that they *“live rurally and were like three hours from anywhere.” (Eleanor, Daughter, MM5, Outer Regional).* This participant reported her parent’s diagnosis process included flying from their regional town to a main city to access an MRI scan.

*“They’ve flown [her] down to [the city] for the day, and she’s had an MRI. She’s come back and finally I get a call saying… ‘[It] might be dementia.’” (Eleanor, Daughter, MM5, Outer Regional).*

Other rural participants with closer proximity to a main regional centre providing health and dementia specialist services weren’t required to travel longer distances. However, inner regional participants were not exempt from lengthy travel, with some participants travelling 150–320 kms to access specialists and tests.

Dementia specialists and services travelling to rural areas was one approach to bridging gaps in access equity. Visiting specialists to rural communities can significantly reduce the travel burden on patients and significant others, with several participants positively describing the benefits of these services. However, many visiting specialists were still relatively difficult to access and participants reported long wait lists, infrequent visits, and high demand. One participant saw a geriatrician who visited the local area during the diagnosis process, and said this was beneficial but the geriatrician only had limited appointment availability. The participant’s other appointments in the main regional centre required a three-hour drive. A few other participants echoed the benefits yet limited appointment availability of visiting specialists.

*“There’s no specialists here. You have to wait weeks to see people. A geriatrician came over who I thought was terrific. He’s the only person that I found in the whole experience who was useful and sensible… But he’s three hours away. He only came over occasionally.” (Abigail, Daughter, MM4, Outer Regional)*

*“That was the other thing, sourcing those kinds of supports… this geriatrician was only coming once a month and he was heavily booked out so that was going to take about three or four months before you could get an appointment.” (Bridgit, Daughter, MM3, Inner Regional)*

Accessing healthcare and dementia specialist services virtually minimised geographical burden as people from rural communities were able to attend some appointments without any travel. Participants in metropolitan settings rarely discussed the use of telehealth as part of their dementia journeys. However, rural participants found telehealth delivered by centralised health teams to be a useful approach to get advice and support on dementia when services weren’t available locally.

*“Jude sees the neuropsychologist … Sometimes [we travel to the city]. Sometimes telehealth.” (Loretta, Partner/Spouse, MM4, Inner Regional)*

*“[Other rural towns had] some dementia support places that they did consult about her not wanting to shower and things like that. I talked to them a couple of times on the phone. They were very good.” (Abigail, Daughter, MM4, Outer Regional)*

Despite this, rural participants also reported many challenges of service coordination delivered by centralised services. Some of the reported disadvantages of non-localised services included feeling the specialists and services had less knowledge of the patient, limited local knowledge, and reduced ability to connect with local services. For example, one daughter described an encounter with a centralised support service who cancelled her mother’s much needed care services. The cancellation occurred after a brief phone consultation by the centralised support service with her mother who had been recently diagnosed with dementia. Her mother told the service she didn’t need support services and they were cancelled as a result, despite being assessed by the government as requiring high-level support. The central organisation’s approach and limited consultation in the decision to cancel the care services created a major barrier to that person’s post-diagnosis care.

*“The person contacted Mum by phone, and Mum indicated that she was fine. She didn’t need anything. She was managing… This person who has not met Mum, who has not spoken to Mum or interviewed Mum, took that as a given and cancelled the whole thing despite being assessed at a Level 4 (having high-level care needs).” (Bridgit, Daughter, MM3, Inner Regional)*

Despite travelling specialists and virtual access to services through telehealth and centralised coordination, rural participants remain heavily dependent on driving to access dementia specialist services. Participants highlighted having limited viable transport alternatives in their rural communities, which also meant that people diagnosed with dementia were particularly concerned about not being able to drive in the future. Not driving had major implications for people living in rural areas to accessing daily necessities and services including food and medications as described by one daughter *“[Mum] couldn’t drive the car… She lived 7 km out of town. She wasn’t taking her medication because she hadn’t been into town to get the prescription.” (Abigail, Daughter, MM4, Outer Regional).*

Several rural participants pointed out that health professionals did not always consider the reliance on driving for transport and access to services and daily activities. One rural participant shared that at the appointment where their partner was told they had dementia, their partner’s licence was taken away immediately, without consideration of the implications and how they would get home. This significant other reported *“[The doctor said,] ‘You can’t drive. [The doctor] did not even know if I had a license for us to be able to drive the hour and a half to get home.” (Loretta, Partner/Spouse, MM4, Inner Regional).*

### Affordability

Several rural participants raised the costs associated with appointments and tests throughout the dementia diagnosis process as barriers to equitable access. This included greater perceived costs associated with travel to various appointments and tests when compared to metropolitan areas, plus any out-of-pocket or private appointments and tests. There are processes available for some rural residents to claim partial reimbursements for travel costs from the government. However, processes for making these travel claims were described as difficult to navigate and required technological proficiency, which presents as another barrier to healthcare access for people going through a dementia diagnosis.

*“You can claim for your travel costs if you’re a rural resident going to the city or within so many kilometres… However, you’ve got to pay for all of that upfront… It’s not even just that. If you are an elderly person who doesn’t have the capacity to scan documents and do everything online… Mum would never have navigated that.” (Glenda, Daughter, MM3, Outer Regional)*

### Availability

As communities decreased in population size and/or distance from main cities grew, participants increasingly described limited access and availability to healthcare and support services for dementia diagnosis, management, and care. Rural participants reported limited numbers of health professionals available in their region and experienced greater difficulty in accessing general practitioners and specialists (e.g., geriatricians, neurologists). Rurality was considered to be a key determinant of access to health services, with a daughter from a medium rural town noting that *“being regional, you don’t have the same access to things.” (Dorothy, Daughter, MM4, Inner Regional)* Participants said that their rural community’s health needs coupled with limited availability of local healthcare staff often resulted in extremely busy and *“really inaccessible*” *(Eleanor, Daughter, MM5, Outer Regional)* health services. Even participants who described their rural community as having good health facilities still faced challenges in having an adequate local health workforce to deliver health services.

Several rural participants described having sufficient availability of some dementia services in their area. The services reported as being more available in rural areas were often post-diagnosis, with adequate home care support providers and residential aged care facilities mentioned in some large rural towns.

*“Look, there’s quite a few aged care facilities around the district. (Glenda, Daughter, MM3, Outer Regional)*

*“There are a few [home care support providers]. The town does have, in terms of demographic, a lot of people over the age of 60. That’s the population. It caters very well. There’s quite a lot of services” (Bridgit, Daughter, MM3, Inner Regional)*

However, other rural participants faced limited local healthcare options for homecare and support services to enable rural people living with dementia to remain at home and finite distribution of post-diagnosis programs, which were more commonly reported in metropolitan communities. Many rural participants felt a disparity in access and choice to healthcare services and support in rural communities compared to those living in metropolitan or larger neighbouring rural communities.

*“I know that there’s a shortage of counsellors and psychologists and psychiatrists. I know there’s tons in [the city] and all that. But there’s very few out this way.” (Morgan, Person with Mild Cognitive Impairment, MM2, Inner Regional)*

### Acceptability

Rural and metropolitan participants’ experience and perceptions about the quality of services and care varied throughout the dementia diagnosis. Acceptability and quality of experiences were attributed to a number of factors; including the variety and accessibility of health professionals, the professional’s expertise and knowledge, their ability to listen and empathise with concerns, how the health professionals communicated test results and the diagnosis, the degree a person’s broader circumstances were considered, and how the person’s care and support needs were considered. Participants frequently described an occasion of service or care as negative when multiple factors were experienced as lacking.

Several rural participants believed that metropolitan services were more acceptable than what they had access to in their rural community. These accounts of differences of acceptability between rural and metropolitan services were often attributed to feeling a lack of choice, experiences of professionals with limited knowledge or empathy to assist with their concerns, or wanting additional specialist expertise and care only available in metropolitan areas. In a rural dyad interview with a person diagnosed with dementia and their spouse, they described how, after the initial diagnosis, they elected to travel further to see a health professional specialising in particular dementias, rather than access services that are geographically closer. The person with dementia verbally and non-verbally concurred with their wife, emphasising that the additional travel was considered worthwhile for the specialised care and support they received.

*“We got onto a [specialist dementia clinic]. Jude sees the neuropsychologist and that through there [despite the further travel]. We don’t have any contact with the [original] memory clinic or the neurologist or anyone anymore… I think just having the emphasis around [specific types of dementia and] being able to guide us within that was helpful.” (Loretta, Partner/Spouse, MM4, Inner Regional)*

One metropolitan daughter reflected that in hindsight that they should have taken their rural parent to the city to access specialists for diagnosis rather than rely on *“local general practitioners that’s probably not their area of specialty.” (Dorothy, Daughter, MM4, Inner Regional)* They felt that their parent would have received a more efficient diagnosis within a metropolitan health system than what they experienced.

Rural participants described having lower expectations of healthcare and services due to limited local options in rural communities. One participant, hesitant to seek care from health professionals they knew professionally, waited six months to see someone not known to them about their dementia symptoms. However, ultimately had no choice but to consult a local practitioner that they knew.

*“I was working with all the [health professionals] and it’s a very small place. I didn’t really want to see one of them because I didn’t want it to impact on my work. I was waiting for [someone] to come from out of the area and I waited for six months and then he didn’t come in the end which was a real blow. I had to see a [health professional] up here.” (Yvonne, Person with Dementia, MM2, Outer Regional)*

Another rural participant described having a very poor experience with a home care company following diagnosis but the limited companies in their area restricted access to alternative support.

*“Maybe we were just a bit unlucky or whether it was the [home care] company we were going through, but there weren’t a lot of companies here to choose from that do that type of work. We were a little bit limited at that stage.” (Glenda, Daughter, MM3, Outer Regional)*

Rural significant others also reported an inability to access services where they felt the staff have sufficient knowledge and understanding of dementia. Despite services claiming to have the necessary skills, one participant regularly found that this was not a reality in practice. This challenge was particularly pronounced for non-Alzheimer’s forms of dementia, where specialised dementia knowledge and tailored care are often even more limited.

*“The biggest frustrations along the way have been the constant fighting for supports that are appropriate. Supports that are consistent. So many [speech] therapists [say] ‘Oh yes, we work with people with dementia’ and ‘We’ve got all this knowledge and experience.’ And then it’s like, ‘I really don’t know what to do.’” (Loretta, Partner/Spouse, MM4, Inner Regional)*

*“Finding people who have both the information and the understanding is incredibly rare. They are very, very few. I can’t tell you how many phone calls I made to aged care agencies, advocates, aged care advocacies, and I’d be going around and around in the loop and here we go again kind of thing.” (Bridgit, Daughter, MM3, Inner Regional)*

### Accommodation

Whilst many participants acknowledged being *“incredibly grateful” (Bridgit, Daughter, MM3, Inner Regional)* for the healthcare systems available in Australia, the systems themselves were often a barrier to accessing services throughout the diagnosis, management, and care process. Both metropolitan and rural participants spoke about the convoluted and fragmented health and social care systems which hindered their ability to enter, navigate, and access the services they needed. Participants reported that in the absence of a pathway to follow, people are forced to *“wait for the next catastrophe” (Ramona, Daughter, MM1, Major Cities of Australia)* to guide dementia care. Many participants described the extensive time and effort required to first determine how to enter and then navigate complex systems (e.g. disability, health, aged care), and the difficulty of these for people living with dementia and their families. A rural location often compounded the complexity to navigating health and social care systems.

*“The systems did me over. Trying to make sense of the requirements. Just trying to understand… It’s just too complicated.” (Bridgit, Daughter, MM3, Inner Regional)*

Rural participants described the inability of people with first-hand experience to guide them through the dementia diagnosis, management, and care process due to the lack of consistency. Each journey was unique with different tests and services involved, meaning one family’s pathway was not necessarily the same as another family despite the same diagnoses. Health professionals struggled to inform patients and significant others of the services available within their rural community, particularly if they were not from that region, forcing significant others to assume the responsibility of seeking out appropriate care with minimal support in some cases.

“*Being in that regional area, we couldn’t get the help… We did a lot of research [into support services] and we’d find one person and then they couldn’t help us. And then they say, ‘Have you tried this person?’ And then we tried them. And then say, ‘Oh no, we can’t help you either’.” (Dorothy, Daughter, MM4, Inner Regional)*

*“[My friend has] fallen into it in a way because she’s running me here and running me there and thinking ahead all the time and making such amazing connections to see what I need. Now if [my friend] was taken out of the equation, I’m not so sure. I think I would be feeling fairly isolated.” (Joyce, Person with Dementia, MM2, Inner Regional)*

### Awareness

Many participants described not being aware of healthcare services and supports available for people living with dementia that would have been helpful, with examples of participants finding out too late about much needed supports. Two daughters described services that would have been valuable to know about at diagnosis but that they didn’t discover until they were no longer relevant. They listed cleaning services, community transport, a carers advocacy support network, and an aged and disability service that supports people through the dementia journey as services that could have allowed their person to remain in their homes for longer or provided much needed support to the significant others had they known of their existence. These rural participants highlighted that insufficient knowledge of accessible services in rural communities led to limited post-diagnostic support that could have been addressed by these existing services.

*“I knew nothing about [the aged and disability service]. We were also not referred there…You just would think somewhere along the line doctors or social workers or anyone would have said [something]… She can get cleaning. She can even get community transport we didn’t know about. She lives 7 kilometres out of town. She could’ve stayed at home for a lot longer if we’d known about all of that.” (Abigail, Daughter, MM4, Outer Regional)*

*“There was an organisation locally that was a carers advocacy support network… But they weren’t very well advertised and I didn’t find out about them… Possibly I would have connected with them. I just didn’t know about them.” (Glenda, Daughter, MM3, Outer Regional)*

Both rural and metropolitan participants described health professionals providing limited information at diagnosis that hindered access to post-diagnostic care. Health professionals may lack knowledge of local services, limiting their ability to provide comprehensive information. Significant others often took responsibility for procuring information to facilitate acceptable dementia diagnosis, management, and care.

*“[My parents] almost became recluses in their own home. They were all self-funded retirees who thought they’d get nothing from the government… They didn’t realise that actually there was some resources around or they didn’t know the process and for myself, as the one who at the end of the day got them through the process and became the aged care expert.” (Alice, Daughter, MM2, Inner Regional)*

However, some rural participants did receive support accessing information and services, such as through a nurse who had seen many families through the diagnosis process. One health service organised a social worker for people diagnosed with dementia who coordinated services to meet the individual’s needs. Others weren’t as fortunate and had to source this information on their own.

*“[My neurologist] did tell me that there was a woman who was a sociologist who was very bright and very across rare dementias. I saw her and she was a wealth of knowledge. She wasn’t squeamish. She wasn’t emotional. She was just very clear. She was able to say there’s this resource, there’s that resource.” (Beatrice, Person with Dementia, MM2, Outer Regional)*

*“They organised a social worker with an intermediary organisation… Joyce can say what she’d like done, whether it’s cleaning or personal care or whatever, and that’s happening twice a week now. All that’s happened within a week and a half of coming out at hospital.” (Jacqui, Friend, MM2, Inner Regional)*

Several participants recommended the establishment of a central location for information in each community that can be the first point of call to ask questions and learn about what is available. A localised service could aid navigation through the complex system and provide support for both the person with dementia and significant others within their community. However, support provision in the rural context presents challenges for viability, with one participant mentioning a similar service used to be available in their rural town but is no longer open.

*“[There needs to be] someone to help [people] navigate through the system however that system looks in their area and in their world. It’s something that supports the family and the people who are supporting the person with the diagnosis.” (Loretta, Partner/Spouse, MM4, Inner Regional)*

*“There is no central place you can go to. There used to be an aged care office in town. You go in and they tell you what all the services were. There’s none of that anymore… [There needs to be] some places where someone can go in an area to ask all the questions they want to ask [and] find out what’s available. I think it’s badly needed.” (Abigail, Daughter, MM4, Outer Regional)*

### Timeliness

Timeliness of dementia services and care was a key challenge for many participants. Lengthy wait times left them with long periods of uncertainty, stress, and anxiety about the future. There was strong consensus that the wait times for specialist help-seeking were much longer than optimal for the best health outcomes. In both rural and metropolitan areas, people undergoing a diagnosis of dementia and their significant others described relatively long waiting times for appointments with specialists and tests, often between three to six months. Level of remoteness didn’t seem to affect this time frame, with long wait times identified irrespective of rurality.

*“[My general practitioner] gave me a referral to [a memory clinic] over in [the nearest regional centre]. I don’t remember how long it was before we actually went to see them. I know it was a while… It was months.” (Morgan, Person with Mild Cognitive Impairment, MM2, Inner Regional)*

*“It took months before [Dad] saw a geriatrician, and I know we can’t fix the delays in this conversation, but it needs to be noted the sparseness of the availability of specialist care and psychological care in such an acute distressing time.” (Harriet, Daughter, MM1, Major Cities of Australia)*

## Discussion

The results of this study highlight that significant inequity in dementia diagnosis, management, and care exist across rural and metropolitan communities in Australia. Disparity in access to health and support services between metropolitan and rural areas was evident across five dimensions: geography impeding ability to access services; affordability of travel expenses; availability of healthcare and support services; acceptability of available health professionals and services; and awareness of local services and resources. Not all healthcare and service inaccessibility were inequitable in relation to location, with affordability of diagnostic tests, lengthy wait times and difficulty navigating complex systems impeding access in both metropolitan and rural communities.

Rural people with dementia and significant others experienced difficulties accessing specialist healthcare and support services, with the extent of difficulty increasing with a person’s remoteness. This is consistent with the experiences of rural people in European, African, North American, Asian, and Oceania studies, where rural people with dementia often have fewer specialist consultations and visit fewer physicians overall compared to metropolitan people with dementia [7, 13, 34]. Difficulties accessing services extended beyond healthcare to support services available, with rural people with dementia and significant others struggling with less choice around amount, type, and provider of services and a lack of support if none were suitable [8]. In this study, both metropolitan and rural people with dementia and significant others faced difficulties around system navigation, particularly in obtaining support and ongoing care in their communities after diagnosis. Qualitative studies conducted in Canada have shown that limited availability of services poses an additional challenge, especially when accompanied by dissatisfaction with the provided care [35, 36]. The perception of less availability and poorer quality of health services in rural communities compared to metropolitan areas is consistent with that reported in a qualitative systematic review of North American, Asian, European, African, and Oceania studies [37].

Geographical constraints are a considerable barrier to accessing healthcare services as people may need to travel significant distances to a metropolitan centre to access specialist services and infrastructure [38]. Reliance on significant others for transport to manage this distance is common amongst people with dementia, with a qualitative study in rural England finding limited transport options contributing to the dependency on others [39]. Rural participants in this study reported traveling 150–550 km, mostly driving, to attend specialist appointments and diagnostic tests. While travel costs were rarely discussed, they were likely significant. Long-distance travel is normalised in rural life with participants emphasising the services accessed over the challenges or expenses of the journey [40].

Accessing appropriate healthcare and support services poses a significant logistical, relationship, and mental challenge for all significant others to navigate amidst complex systems with lengthy wait times [35, 41]. Median diagnostic wait times for dementia and Mild Cognitive Impairment in Australia are reported to be 4.2 months from primary physician/general practitioner referral to memory or cognitive clinic to diagnosis; however, the impact of rurality on these time intervals is unclear [42]. These challenges are not unique to dementia, with rurality impacting health outcomes irrespective of health condition [12, 43]. Increased access to dementia-specific services in rural regions and examination on the role of rurality on accessibility is a priority to reduce the inequity between rural and metropolitan dementia diagnosis, management, and care experiences [44, 45].

As innovative diagnostic tests, symptom management drugs, and disease-modifying therapies are introduced into clinical practice, it is imperative that inequities in the Australian healthcare system are understood and mitigated. Without awareness and purposeful action, the implementation of these advancements risk widening the disparities between rural and metropolitan areas. Disease-modifying therapies require specialist care to regularly administer the medication regime and involve comprehensive monitoring for adverse effects [46]. Workforce shortages and finite resources limit capacity to provide structured dementia care in rural areas [12] and rural participants may need to routinely travel to regional centres or metropolitan areas for treatment. Novel diagnostic tests have the potential to improve access to quality dementia care irrespective of location. For example, advancements continue to be made to produce safe and reliable blood biomarker measures for accurate diagnosis of Alzheimer’s disease [47]. Blood biomarkers may present an opportunity to improve access as they require less specialised infrastructure, such as access to lumbar puncture or PET facilities [47]. Strategies to minimise the burden on rural people with dementia and significant others will be critical to promote equitable dementia diagnosis, management, and care.

Capitalising on technological advancements to diminish the impact of immense distances can also alleviate some of the strain experienced by vulnerable individuals provided there is a focus on building relationships, trust, and understanding the local context for rural people living with dementia [48]. A systematic review with studies from Australia, Canada, Korea, and United States suggested that geographical isolation and logistical load can potentially be reduced by telehealth applications to facilitate diagnosis and ongoing care [49]. Digital cognitive assessments have been shown to have comparable or improved diagnostic performance to traditional tests whilst increasing accessibility, reducing health professional’s workload, and facilitating monitoring of performance [50]. While digital health solutions present an opportunity to bridge the disparity between rural and metropolitan communities, a scoping review of studies from North America, South America, Asian, Europe, Africa, and Oceania revealed significant challenges to access [51]. Many rural communities lack the infrastructure for reliable internet connectivity, households may not have access to the requisite technology, and individuals could possess limited digital literacy [51]. As society enters into the new dementia care paradigm, research and investment must be made into interventions that improve accessibility for all and reduce inequalities.

The Dimensions of Access [33] provided a systematic approach to examine the concept of access in the context of rural and metropolitan experiences with dementia diagnosis, management, and care. This framework allowed accessibility to be anatomised to compare rural and metropolitan experiences to ascertain inequity between the two contexts and dimensions requiring improvement for all Australians. This framework enabled us to delve into contextual and compositional factors affecting access. Methodological evaluation of the current dementia context in Australia is crucial to optimise dementia diagnosis, management, and care and address differences in healthcare access and outcomes between rural and metropolitan areas. However, rural participants did not always detail the impact of their rurality, and there is greater opportunity for targeted research to further explore diagnosis journeys for those in regional, rural, and remote areas of Australia. Embedding the Dimensions of Access framework could inform future research on the aspects of rural life that influence health equity.

This study comprised of a mixed group of people diagnosed with dementia and significant others with varying diagnoses, age at diagnosis, years since diagnosis and state/territory of diagnosis. There were uneven groupings amongst rural and metropolitan participants, with a greater proportion of people with dementia and diagnoses at ages less than 65 years in the rural group compared to the metropolitan participants. An important limitation of this study is that we were unable to recruit participants living in remote or very remote areas of Australia, which means that their perspectives and experiences with accessibility during dementia diagnosis, management, and care were not captured in this study. Further, only participants able to communicate in English were included in this study, and it will be important for future studies to specifically explore the experiences of individuals from culturally and linguistically diverse backgrounds. Additionally, experiences and communities are not homogenous, with findings of this study not necessarily reflective of the experience of other people with dementia and their significant others. During descriptions of dementia journeys, participants tended to provide great detail on the negative aspects of their experience and omit the more positive or neutral elements. Significant others were not always actively involved throughout the entire dementia journey. Some significant others did not live nearby when symptoms first presented, or they assumed responsibility only when another significant other was no longer able to coordinate the provision of care. As this study describes accounts of the diagnosis, management, and care experience, which occurred over a wide timespan and with varied type of participants, some accounts and reflections may also be impacted by challenges with recall. The absence of a specified timeframe for when participants experienced the diagnosis process may impact the comparability of their experiences, as those diagnosed up to 20 years ago could have different experiences with diagnosis and post-diagnostic support compared to those diagnosed more recently.

## Conclusion

Disparities in access to health and social care in rural communities negatively impacts access to diagnosis, management, and care for people with dementia and their significant others in Australia. Examining dementia diagnosis, management, and care experiences according to the Dimensions of Access provides a framework to identify inequities and develop targeted strategies to promote quality dementia care for all. As advances in dementia research related to diagnostics and treatments are increasingly translated into clinical practice, it is crucial that mitigating the impact of rurality is prioritised to promote equitable dementia diagnosis, management, and care.

## Supplementary Information

Additional file 1.

Additional file 2.

## Data Availability

The data generated during the current study are not publicly available because study participants did not consent to the data being made available to other researchers. However, we have provided the interview guides used to collect data for this study (see Additional files 1 and 2). Furthermore, additional qualitative data from our dataset are being prepared for publication elsewhere.

## Abbreviations

ASGS-RA: Australian Statistical Geography Standard Remoteness Areas
MMM: Modified Monash Model
ARIA +: Accessibility/Remoteness Index of Australia Plus
