# Supplementary material for: Equity of access in rural and metropolitan dementia diagnosis, management, and care experiences: an exploratory qualitative study
Source: Int J Equity Health. 2025 Mar 17;24:74. doi: 10.1186/s12939-025-02434-1 (PMC11912628; doi:10.1186/s12939-025-02434-1)
Supplement: Supplementary file 1 — Additional file 1. [file 12939_2025_2434_MOESM1_ESM.docx]

**Additional File 1 - Patient Interview Guide**

1. **I’d like to start by asking a few things about you.**
   1. A fun question about you……what is a favourite place you have lived, worked or travelled to?
   2. Your important activities

For example, that might include social activities and groups, work and volunteer work, learning and study, caring for others.

1. **Can you please tell me about you and your family?**
2. Where you live and a bit about the people in your household
3. And your extended family members
4. Do you or any members of your family speak different languages?
5. And thinking about different cultures throughout Australia, can you please tell me a bit about your cultural background?

As part of the interview discussion we will be drawing a river that represents your dementia diagnosis journey. There is no right or wrong way to do this activity – each River of Life drawing will be different.

The River of Life drawing will be created as part of the interview discussion. It can be a helpful way to recall all the things that happened along your dementia diagnosis journey. The interviewer will use an online program to create your River of Life drawing, which you will receive a copy.

**Let’s start the questions about your dementia diagnosis journey.**

1. **When did the signs or symptoms of dementia first start to appear?**
   1. What year (how many years ago) did they start to appear?
   2. What age did the signs and symptoms start to appear?
   3. What were the signs and symptoms that appeared?
   4. Who noticed these signs and symptoms?
   5. What actions or steps were taken at this time?
   6. How did you feel?

| **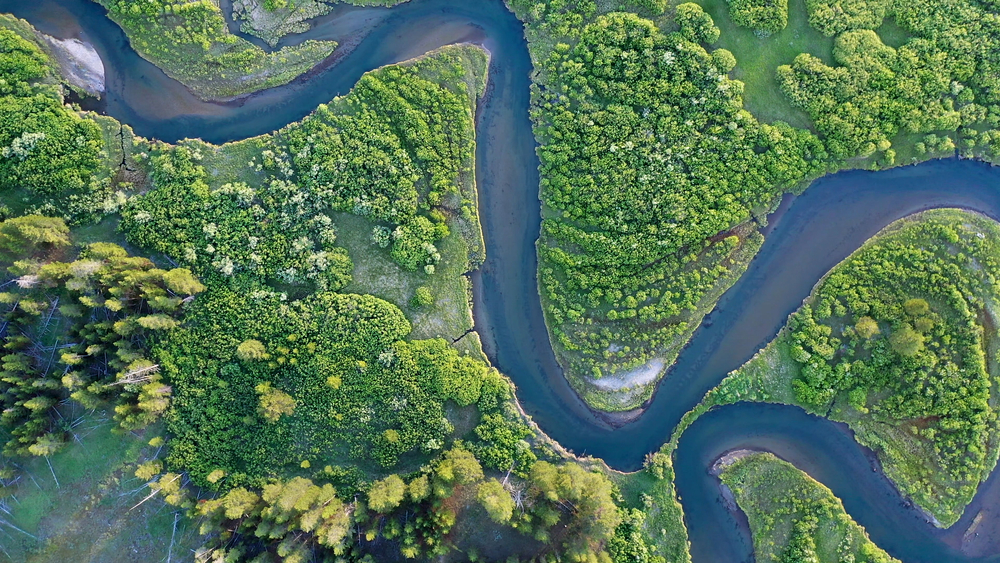** | **Step 1. Mapping the River of Life for the diagnosis journey: the start of the river**   - How would you describe your dementia diagnosis journey if it was a river? - What does it look like? - Who is at the start of the river? - Does the river flow fast or slow? |
| --- | --- |

1. **What health professionals or other professional/s did you see in seeking help for the signs and symptoms you were experiencing? E.g. GP, specialist.**
   1. Who did you see? When was this?
   2. How long did it take to get appointments to see the health and other professionals?
   3. Was it difficult to get to or access the appointments? How far did you have to travel to attend appointments?
   4. What happened at the appointments with the health and other professionals?
   5. Did you get information from health or other professionals? If so, was the information you received helpful?
   6. How did you feel?
2. **What tests did you have to explore the signs and symptoms you were experiencing?**
   1. Who did the tests?
   2. When were the tests done?
   3. How clearly were the tests explained?
3. What information did you receive after having the tests? How helpful was the information?

|  |  |  |
| --- | --- | --- |

1. **When did you first find out that the signs and symptoms you were experiencing were dementia?**
   1. What happened at this time?
   2. Who confirmed the diagnosis?
   3. How long was after the first signs and symptoms was the diagnosis confirmed?
   4. How did you feel?
2. **Who else was involved in supporting you during your process of being diagnosed?**
3. Family, friends, work colleagues, other people or organisations?

| **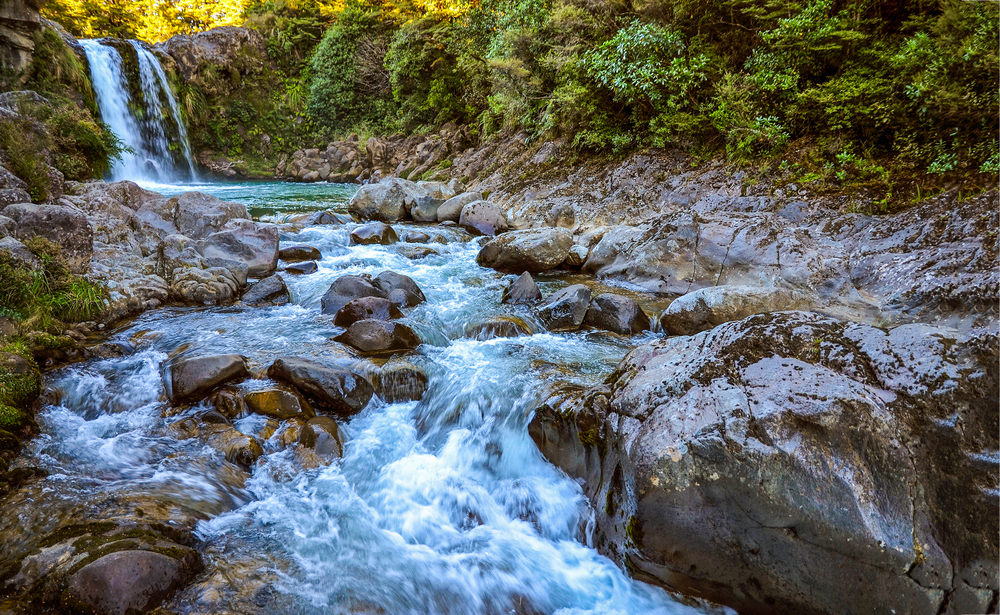**  **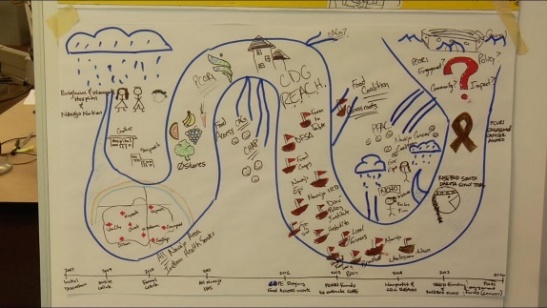** | **Step 2. Mapping the River of Life: what happens along the river?**   - What does it look like along the river? - When are there changes in the river? (when the situation or perspective changed) - Does the river have sudden changes or bends? Is your river long and winding? - Does the river have any boulders, rapids or waterfalls? - What does the river look like at the time of getting the diagnosis? |
| --- | --- |

1. **What did you find was unhelpful or difficult in going through the dementia diagnosis process?**
2. **What was helpful or made it easier in going through the dementia diagnosis process?**
3. The people, health or other professionals involved
4. Services and supports
5. Information and resources
6. **What would you like to see changed in dementia diagnosis?**
7. **Before we finish, is there anything else you would like to add?**
